# Lucidone suppresses dengue viral replication through the induction of heme oxygenase-1

**DOI:** 10.1080/21505594.2017.1421893

**Published:** 2018-02-27

**Authors:** Wei-Chun Chen, Chin-Kai Tseng, Chun-Kuang Lin, Shen-Nien Wang, Wen-Hung Wang, Shih-Hsien Hsu, Yu-Hsuan Wu, Ling-Chien Hung, Yen-Hsu Chen, Jin-Ching Lee

**Affiliations:** a Graduate Institute of Medicine, College of Medicine, Kaohsiung Medical University, Kaohsiung, Taiwan; b Institute of Basic Medical Sciences, College of Medicine, National Cheng Kung University, Tainan, Taiwan; c Center of Infectious Disease and Signaling Research, College of Medicine, National Cheng Kung University, Tainan, Taiwan; d Doctoral Degree Program in Marine Biotechnology, College of Marine Sciences, National Sun Yat-Sen University, Kaohsiung, Taiwan; e Division of Hepatobiliary Surgery, Department of Surgery, Kaohsiung Medical University Hospital, Kaohsiung Taiwan; f Department of Surgery, Faculty of Medicine, Kaohsiung Medical University Hospital, Kaohsiung Taiwan; g Department of Internal Medicine, Kaohsiung Medical University Hospital, Kaohsiung, Taiwan; h Division of Infectious Diseases, Department of Internal Medicine, Kaohsiung Medical University Hospital, Kaohsiung, Taiwan; i School of Medicine, Graduate Institute of Medicine, Sepsis Research Center, Center for Dengue Fever Control and Research, Kaohsiung Medical University, Kaohsiung, Taiwan; j Department of Biological Science and Technology, College of Biological Science and Technology, National Chiao Tung University, HsinChu, Taiwan; k Center for Infectious Disease and Cancer Research, Kaohsiung Medical University, Kaohsiung, Taiwan; l Department of Biotechnology, College of Life Science, Kaohsiung Medical University, Kaohsiung, Taiwan; m Graduate Institute of Natural Products, College of Pharmacy, Kaohsiung Medical University, Kaohsiung, Taiwan; n Research Center for Natural Products and Drug Development, Kaohsiung Medical University, Kaohsiung, Taiwan; o Department of Medical Research, Kaohsiung Medical University Hospital, Kaohsiung, Taiwan

**Keywords:** Dengue virus, lucidone, heme oxygnase-1, viral replication, interferon response

## Abstract

Dengue virus (DENV) infection causes life-threatening diseases such as dengue hemorrhagic fever and dengue shock syndrome. Currently, there is no effective therapeutic agent or vaccine against DENV infection; hence, there is an urgent need to discover anti-DENV agents. The potential therapeutic efficacy of lucidone was first evaluated *in vivo* using a DENV-infected Institute of Cancer Research (ICR) suckling mouse model by monitoring body weight, clinical score, survival rate, and viral titer. We found that lucidone effectively protected mice from DENV infection by sustaining survival rate and reducing viral titers in DENV-infected ICR suckling mice. Then, the anti-DENV activity of lucidone was confirmed by western blotting and quantitative-reverse-transcription-polymerase chain reaction analysis, with an EC_50_ value of 25 ± 3 μM. Lucidone significantly induced heme oxygenase-1 (HO-1) production against DENV replication by inhibiting DENV NS2B/3 protease activity to induce the DENV-suppressed antiviral interferon response. The inhibitory effect of lucidone on DENV replication was attenuated by silencing of HO-1 gene expression or blocking HO-1 activity. In addition, lucidone-stimulated nuclear factor erythroid 2-related factor 2 (Nrf2), which is involved in transactivation of HO-1 expression for its anti-DENV activity. Taken together, the mechanistic investigations revealed that lucidone exhibits significant anti-DENV activity in *in vivo* and *in vitro* by inducing Nrf2-mediated HO-1 expression, leading to blockage of viral protease activity to induce the anti-viral interferon (IFN) response. These results suggest that lucidone is a promising candidate for drug development.

## Introduction

Dengue virus (DENV) is an arthropod-borne pathogen causing a human viral disease that spreads via infected mosquito vectors across most of the tropical and subtropical world [1]. About 400 million people are infected with DENV and 2.5 billion people are at risk of infection. DENV is an enveloped virus in the *Flaviviridae* family. It has an 11-kb RNA genome that is translated into a polyprotein and is subsequently cleaved by both host and viral proteases into structural proteins (C, prM, and E) and non-structural proteins (NS1, NS2A, NS2B, NS3, NS4A, NS4B, and NS5) [2]. DENV is divided into four different serotypes (DENV-1–4) based on the antigenic diversity, which have been associated with the clinical manifestations of classical dengue fever, severe dengue hemorrhagic fever (DHF), and dengue shock syndrome (DSS) [3]. Today, there are tetravalent dengue vaccines with limited efficacy but no approved drugs against DENV infection. Hence, there is still a need to discover potential viral or host targets for anti-DENV drug development.

Previous studies have reported a significant correlation among DENV infection, cellular oxidative stress, and several reactive oxygen species (ROS)-scavenging molecules, including heme oxygenase-1 (HO-1), which is beneficial in alleviating the risk of oxidative stress-related diseases [4,5]. HO-1 is an inducible enzyme in the heme catabolic pathway and one of the protective enzymes produced to reduce oxidative stress that catalyzes the degradation of heme into biliverdin, carbon monoxide, and ferrous iron, which play important roles in cytoprotection [6,7]. HO-1 induction has been reported to be a promising strategy against DENV replication by suppressing DENV NS2B/3 protease activity, leading to the stimulation of DENV-reduced anti-viral IFN responses [5]. These findings indicate that HO-1 can be considered a potential therapeutic target in DENV therapy.

*Lindera erythrocarpa* Makino, widely cultured in Asian countries, belongs to the family Lauraceae and its fruits are used as a folk medicine because of the extensive pharmacological actions, including analgesic, antibacterial, antidotal, diuretic and digestive activities [8]. Lucidone, is a phytochemical isolated from the fruit of *L. erythrocarpa* that exerts anti-inflammatory activity with significant suppression of inducible nitric oxide synthase (iNOS) and cyclooxygenase-2 (COX-2) production [9]. In our previous study, we reported that lucidone markedly inhibits hepatitis C virus (HCV) replication by inducing HO-1 expression, leading to enhancement of the antiviral IFN response and inhibition of viral protease activity [10]. In the present study, we investigated the antiviral effect on DENV replication and the protective effects of lucidone on ICR suckling mice against the life-threatening DENV-2 infection and further address the possible mechanism of lucidone activity against DENV replication.

## Materials and Methods

### 1.1. Ethics statement

The breeder mice of the ICR strain were obtained from BioLasco Taiwan Co. Ltd. and acclimatized under standard laboratory conditions following the Animal Use Protocol of Kaohsiung Medical University for a week before the experiment. All procedures and protocols were approved by the Animal Care and Use Committee of Kaohsiung Medical University (IACUC, 102177). The mice were maintained at the Animal Facility of Kaohsiung Medical University and were manipulated according to the guidance of the Public Health Service Policy on Humane Care and Use of Laboratory Animals. The virus used in animal model study is DENV type 2 strain PL046 which was kindly provided by Dr. Huey-Nan Wu (Institute of Molecular Biology, Academia Sinica, Taipei, Taiwan) [11].

### 1.2. Cell culture and reagents

Huh-7 (kindly provided by Dr. Charles Rice, Rockefeller University and Aapth, LCC, USA) and BHK-21 (purchased from ATCC, Manassas, VA, USA) cells were both maintained in Dulbecco's Modified Eagle's Medium supplemented with 10% heat-inactivated fetal bovine serum, 1% antibiotic-antimycotic, and 1% nonessential amino acids (The components were purchased from GIBCO/Invitrogen, Germany). All cells were incubated at 37 °C with a 5% CO2 supplement. Lucidone was purchased from Chem Faces (CFN98011). Tin protoporphyrin IX dichloride (SnPP; P8293) was obtained from Sigma-Aldrich Co. The chemical agents were prepared as a stock solution at 100 mM in 100% DMSO. The final concentration of DMSO in the all the experiments was constantly maintained at 0.1%. HO-1 (NM_002133), Nrf2 (NM_006164), and enhanced green fluorescent protein (EGFP) small hairpin RNAs (shRNAs) were purchased from the National RNAi Core Facility, Institute of Molecular Biology/Genomic Research Center, Academia Sinica, Taiwan. These specific shRNAs were used to confirm the mechanism underlying the lucidone-mediated anti-DENV activity. All DNA fragments were confirmed by DNA sequencing.

### 1.3. DENV infection of mice

The breeder mice of the ICR strain were purchased from BioLasco Taiwan Co. Ltd. Six-day-old ICR mice were intracranially inoculated with active or heat-inactivated DENV type 2 strain (2.5 × 10^5^ pfu per mouse) medium containing 2% FBS at day 1. Subsequently, the mice were intracranially injected with lucidone or saline at day 2, 4, 6. The body weight, evaluate clinical signs, and mortality of mice were monitored and recorded daily for 7 days (n ≥ 8/group). Clinical symptoms were scored as follows: 0: healthy; 1: weight loss, ruffled hair, or hunchbacked appearance; 2: reduced mobility; 3: moving with difficulty and limb weakness; 4: limb paralysis; and 5: moribund or death.

### 1.4. Plaque assay

The infectious viral particles were isolated from the brain of the DENV-2 infected mice. BHK-21 cells at the density of 9 × 10^4^ cells per well were infected with serially diluted virus solutions for 2h. The infected cells were replaced with fresh DMEM containing 2% FBS and 0.8% methyl cellulose (Sigma-Aldrich, 274429). At 4 days postinfection, the medium was removed and washed with PBS. Subsequently, the infected cells were fixed and stained with the crystal violet solution (1% crystal violet, 0.64% NaCl, and 2% formalin, which were purchased from Sigma-Aldrich) at room temperature. After 1 h, the crystal violet solution was removed and the plate was washed with tap water. Finally, the viral titer was determined by the plaque assay.

### 1.5. Western blot assay

The standard procedure of western blotting was performed as described previously [12]. The membranes were probed with monoclonal antibodies specific for DENV NS2B, (1:5000; GeneTex, GTX124246), glyceraldehyde-3-phosphate dehydrogenase (GAPDH) (1:10000; GeneTex, GTX100118), Nrf2 (1:3,000; GeneTex, GTX55732), HO-1 (1:3,000; Abcam, ab137749), Keap1 (1:1,000; Abcam, ab196346), and Bach1 (1:1,000; Abcam, ab115210). The ECL detection kit was used for signal detection (PerkinElmer, CT, USA).

### 1.6. Quantitative real-time RT-PCR (qRT-PCR) analysis

The total RNA of the cells was isolated using a total RNA miniprep purification kit (GMbiolab Co., Ltd., Taiwan, TR01), according to the manufacturer's instructions. cDNA synthesis was performed using M-MLV Reverse Transcriptase (Promega, M1701), according to the manufacturer's instructions. The levels of DENV NS5, HO-1, IFN-α-2, IFN-α-5, IFN-α-17, OAS family (OAS1-3), PKR and HO-1 RNA were detected by qRT-PCR with specific primer sets as shown in Table 1. The relative RNA levels of these genes in each sample were normalized to cellular GAPDH mRNA. The relative expression levels were analyzed using the ABI Step One Real-Time PCR-System in the standard procedure (ABI, 4309155).

**Table 1.** Oligonucleotide sequences for real-time RT-PCR.

Oligonucleotide Name	Sequence 5′-3′
DENV serotypes	
5′ NS5 (Type1)	5′-CAGGTCAAACGCAGCTATTG
3′ NS5 (Type1)	5′-CCACTCCACTGAGTGAATTC
5′ NS5 (Type2)	5′-TGTATGCCGATGACACCGCA
3′ NS5 (Type2)	5′-TCTTTGCACACGGACCACCT
5′ NS5 (Type3)	5′-TCAGAACTAACGCAGCCATG
3′ NS5 (Type3)	5′-AGAGTTTTCACGCGAGAACC
5′ NS5 (Type4)	5′-AGATCAAACGCAGCCATAGG
3′ NS5 (Type4)	5′-CTTCCACTCCACTCCATGAA
Human factors	
5′ GAPDH	5′-GTCTTCACCACCATGGAGAA
3′ GAPDH	5′-ATGGCATGGACTGTGGTCAT
5′OAS1	5′- CAAGCTTAAGAGCCTCATCC
3′OAS1	5′- TGGGCTGTGTTGAAATGTGT
5′OAS2	5′- ACAGCTGAAAGCCTTTTGGA
3′OAS2	5′- GCATTAAAGGCAGGA AGCAC
5′OAS3	5′- CACTGACATCCCAGACGATG
3′OAS3	5′- GATCAGGCTCTTCAGCTTGG
5′PKR	5′- ATGATGGAAAGCGAACAAGG
3′PKR	5′- GAGATGATGCCATCCCGTAG
5′IFN-α2	5′- GCAAGTCAAGCTGCTCTGTG
3′IFN-α2	5′- GATGGTTTCAGCCTTTTGGA
5′IFN-α17	5′-AGGAGTTTGATGGCAACCAG
3′IFN-α17	5′-CATCAGGGGAGTCTCTTCCA
Mouse factors	
5′ GAPDH	5′-CCATGCCATCACTGCCACCC
3′ GAPDH	5′-GCCATGCCAGTGAGCTTCCC
5′OAS1	5′-GGACCCCGCTGACCCAACAA
3′OAS1	5′-CAACCTCCGTCGGCACCTCC
5′OAS2	5′-GGTGGGTCTTCAGGGGTGCC
3′OAS2	5′-GCAGCAGGTCCCAGATGGCA
5′OAS3	5′-CCGTGCCTGGACTGAGCCTC
3′OAS3	5′-GGCTGAGGTTTGGTGCCGGA
5′PKR	5′-AGAGCCCGCCGAAAACTGCC
3′PKR	5′-CGCTGTTAAACCTGGCGTCCA
5′IFN-α2	5′-TGACCTCCACCAGCAGCTCA
3′IFN-α2	5′-TCTGCTCTGACCACCTCCCA
5′IFN-α5	5′-CCATCCCTGTCCTGAGTGAGCT
3′IFN-α5	5′-AGATTCCTGCACCCCGACCT

### 1.7. Cytotoxicity assay

The relative cell viabilities were determined by the CellTiter 96® AQueous Non-Radioactive Cell Proliferation Assay [3-(4,5-dimethylthiazol-2-yl)-5-(3-carboxymethoxyphenyl)-2-(4-sulfophenyl)-2H-tetrazolium, inner salt; MTS] that depended on the measurement of mitochondrial dehydrogenase enzyme activity from viable cells (Promega, G3582). DENV-2-infected cells at the density of 5 × 10^3^ cells per well were treated with lucidone at different concentrations. After a 3-day incubation period, the supernatant was removed and added to the MTS mixture containing 100 μl of phenol red-free medium and 20 μl of the MTS reagent at 37°C. After 4 h, the relative cell viabilities were analyzed by measuring the absorption at 490 nm on a microplate reader.

### 1.8. DENV infection assay

Huh-7 cells at the density of 5 × 10^4^ cells per well were infected with DENV at a multiplicity of infection of 0.1 for 2 h. At the end of the infection, the supernatant was removed and the cells were incubated with various concentrations of lucidone for an additional 3 days. The total RNA of the cells was isolated and the relative DENV RNA levels were analyzed by the ABI Step One Real-Time PCR-System. The EC_50_ value was analyzed by CalcuSyn software (Biosoft, Cambridge, UK).

### 1.9. Transient transfection and luciferase activity assay

All the transfection reactions were performed using a T-Pro™ reagent (Ji-Feng Biotechnology Co. Ltd., Taiwan, JT97-N001M), in accordance with the manufacturer's instructions. Huh-7 cells at the density of 5 × 10^4^ cells per well were transfected with pHO-1-Luc, p3xARE-Luc, or pISRE-Luc. pHO-1-Luc contains a human HO-1 promoter-driving firefly luciferase expression. p3xARE-Luc, containing three repeats of the Nrf2-dependent antioxidant response element (ARE)-driving firefly luciferase expression, was used to detect the translocation and transcription activity of Nrf2. pISRE-Luc, containing an IFN-stimulated response element (ISRE) driving firefly luciferase expression, was used to detect the activity of IFN response-dependent transcription. The reporter plasmid transfected cells were infected with DENV-2 and then treated with the indicated concentrations of lucidone. The reporter activity was performed and analyzed as previously described [10].

### 1.10. DENV protease activity assay

The protease activity was performed and analyzed as previously described [5]. After incubation for 3 days, luciferase activities within cell supernatant and lysates were analyzed using Nano-Glo (N1110) and Bright-Glo (E2620) Luciferase assay systems (Promega), respectively. To normalize each transfection efficiency, the cells were co-transfected with 0.1 μg of firefly luciferase expression vector (pCMVLuc), and the reporter activity was normalized according to the firefly luciferase activity.

### 1.11. Statistical analysis

Results are presented as the mean ± SD of least three independent experiments. Statistical data comparisons were done by the Student's t-test and ANOVA using GraphPad software. * <0.05, **<0.001, or *** <0.0001 was considered as statistically significant.

## Results

### Physiopathological changes in DENV-infected ICR suckling mice after lucidone treatment

**Figure 1.**
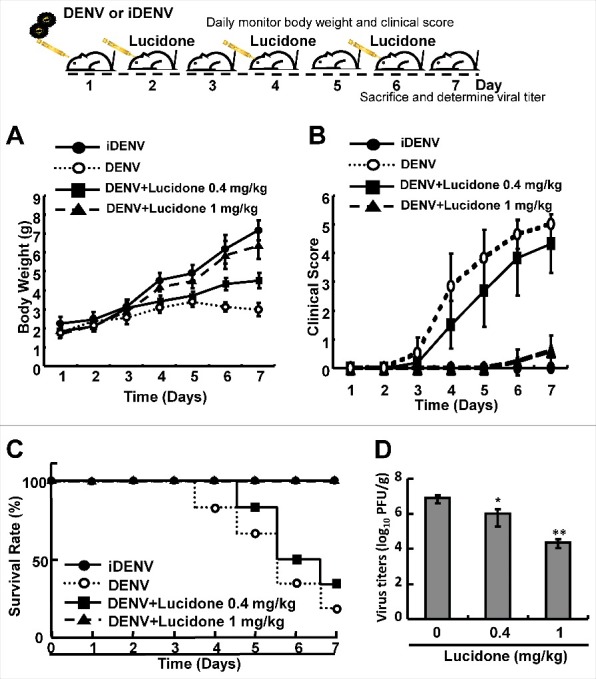
Protective effect of lucidone on DENV-infected ICR mice. The experimental procedure of DENV-infected ICR mice model. Six-day-old ICR suckling mice were divided into three groups, including mice that were intracranially inoculated with DENV (2 × 10^5^ pfu per mouse) with or without lucidone treatment (0.4 or 1 mg/kg) at 2, 4, and 6 days. Mice were intracranially inoculated with 60°C heat-inactive DENV (iDENV) and saline treatment as a negative control. The (A) body weight (B) clinical score (C) survival rate were monitored daily and (D) virus titer was determined on day 7 by plaque assay. Illness symptoms were scored as described in the Materials and Methods section. Each group included more than eight mice. *P versus non-lucidone treated control group.

To identify the protective effect of lucidone on DENV infection *in vivo*, the infectious DENV or heat-inactivated DENV (iDENV) was intracranially injected into 6-day-old ICR suckling mice. The body weight, clinical score, and survival rate of the infected mice were measured daily. As shown in Fig. 1A, no significant decrease was observed in the body weight of DENV-infected mice treated with1 mg/kg lucidone compared to that of iDENV-infected mice. In contrast, DENV-infected mice lost body weight gradually from days 4 to 7. In contrast, the body weight of DENV-infected mice with lucidone treatment obviously increased gradually until day 7. The clinical score of DENV-infected mice treated with lucidone (1 mg/kg) indicated slight disease symptoms on day 7 compared to those in the iDENV-infected group. Notably, lucidone treatment (1 mg/kg) significantly increased the survival rate of DENV-infected mice on day 7 compared to that of untreated DENV-infected mice. Similarly, DENV-infected mice treated with 0.4 mg/kg lucidone showed a reduced survival rate, which delayed the occurrence of disease symptoms and prolonged survival rate (Fig. 1B and 1C). To further investigate the effect of lucidone on viral propagation, DENV-infected mice were sacrificed and their brain tissues were collected to measure the viral titer by plaque assay. As shown in Fig. 1D, the DENV-infected mice with 0.4 mg/kg and 1 mg/kg lucidone treatment showed 0.9-fold and 2-log reduction of virus titer in DENV-infected mice compared to that in untreated mice, respectively (Supplementary Fig. S1).

### Lucidone suppresses DENV replication

**Figure 2.**
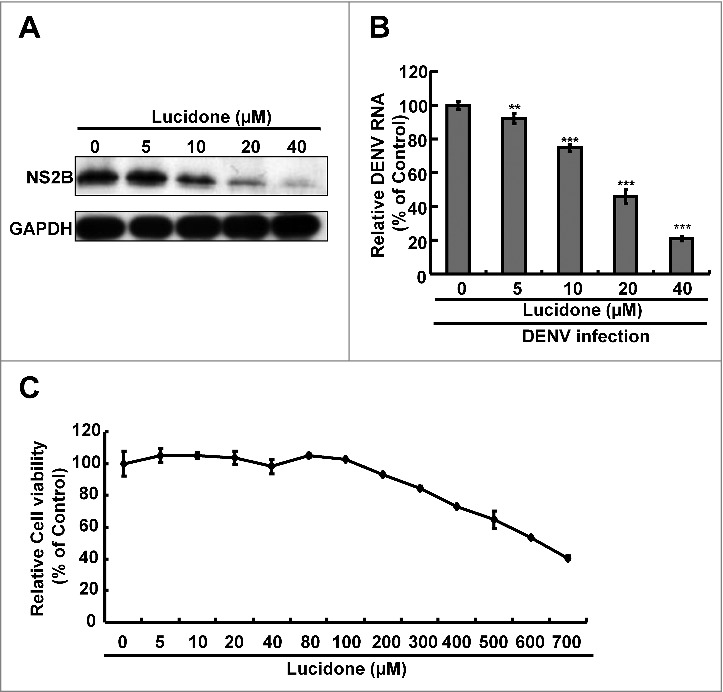
Effect of lucidone on DENV protein expression and RNA replication in DENV-infected cells Lucidone suppressed DENV replication in DENV-infected Huh-7 cells. DENV-infected Huh-7 cells were exposed to lucidone at different concentrations for 3 days. (A) Western blotting was performed using anti-DENV NS2B antibody and anti-GAPDH antibody. GAPDH protein levels showed equal loading of cell lysates. (B) Total RNA of lucidone-treated cells was extracted to quantify DENV RNA levels by qRT-PCR. Relative DENV RNA levels were normalized by cellular *gapdh* mRNA. (C) Cellular toxicity was simultaneously evaluated by the MTS assay. “0” indicates treatment with 0.1% DMSO. DENV RNA levels and cell viability were presented as percentage changes compared to those in lucidone-untreated DENV-infected cells, in which the relative RNA level was presented as 100%. Results are expressed as means ± SD (error bar) of three independent experiments. *P versus non-lucidone treated control group.

The cell-based DENV infectious system was further used to assess the antiviral activity of lucidone on DENV replication. Huh-7 cells were infected with DENV, and then treated with lucidone at a concentration range of 0–40 μM for 3 days. As shown in Fig. 2A, lucidone significantly inhibited DENV protein synthesis in a concentration-dependent manner. Similarly, lucidone reduced DENV RNA levels in a concentration-dependent manner (Fig. 2B) and exhibited an EC_50_ value of 25 ± 3 μM. The cell viability assay showed no obvious cell death at an effective concentration of 40 μM (Fig. 2C). Nevertheless, lucidone also significantly inhibited DENV serotypes 1–4 (Supplementary Fig. S2). Taken together, these findings suggest that lucidone is an effective agent against DENV infection *in vitro* and *in vivo*.

### Lucidone exerts its antiviral activity by increasing DENV-suppressed HO-1 expression upon virus infection

**Figure 3.**
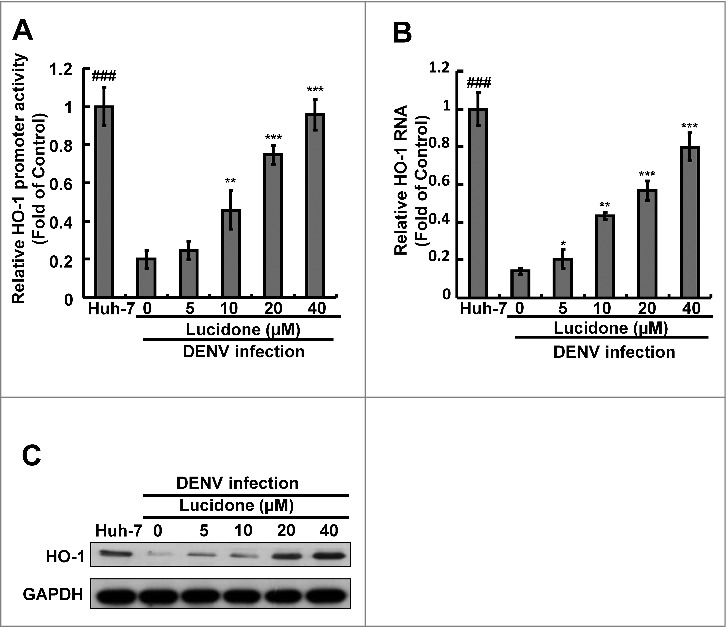
Involvement of the induction of HO-1 expression in the inhibitory effect of lucidone on DENV replication. Lucidone activated DENV-reduced HO-1 expression in DENV-infected Huh-7 cells. (A) Lucidone induced HO-1 promoter activity. Huh-7 cells were co-transfected with the HO-1 promoter reporter vector pHO-1-Luc and pSEAP reporter vector, an internal control. The transfected cells were infected with DENV and treated with lucidone for 3 days, and total cell lysates were analyzed for luciferase activity followed by normalization of SEAP activities. (B, C) Lucidone induced HO-1 expression in DENV-infected cells. Total RNA and cell lysates of Huh-7 and DENV-infected Huh-7 cells with or without lucidone treatment were extracted to analyze HO-1 expression by qRT-PCR and western blotting. Relative HO-1 RNA levels were normalized by cellular *gapdh* mRNA. Western blotting was performed using anti-HO-1 and anti-GAPDH antibodies. The‘0’ indicates treatment with 0.1% DMSO. HO-1 RNA levels were presented as percentage changes compared to parental Huh-7 cells, in which level was presented as 1. Results are expressed as means ± SD (error bar) for three independent experiments. *P versus non-lucidone treated control group; #P versus DENV-infected control group.

**Figure 4.**
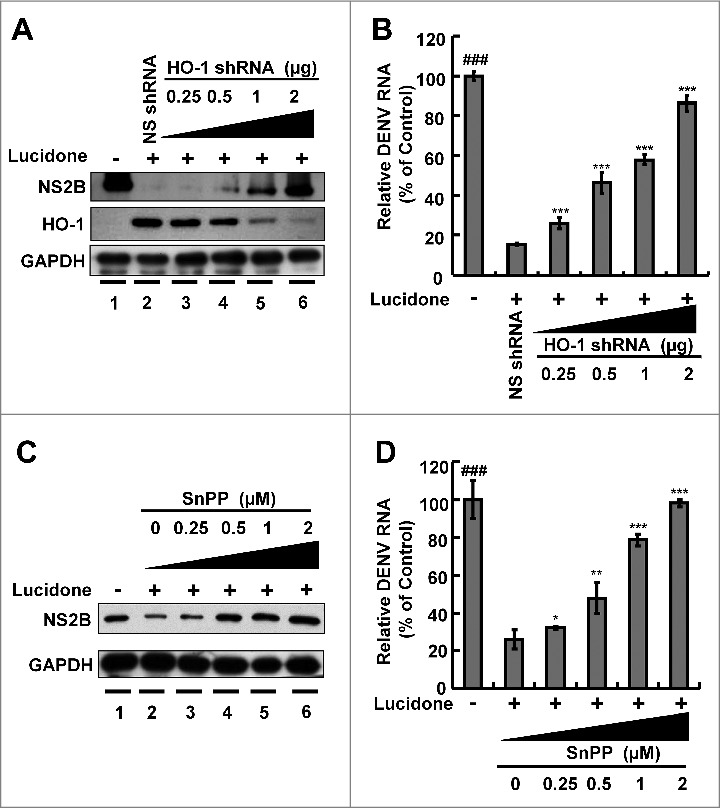
Attenuation of anti-DENV activity of lucidone by inhibition of HO-1 expression and activity. (A, B) Silencing HO-1 expression attenuated the antiviral activity of lucidone. DENV-infected Huh-7 cells were transfected with different amounts of HO-1-specific shRNA (0.25–2 μg) or nonspecific shRNA, and then treated with or without lucidone (40 μM) for 3 days. (C, D) HO-1 inhibitor attenuated the antiviral activity of lucidone. DENV—infected Huh-7 cells were treated with different concentrations of SnPP (0–20 μM) with or without lucidone (40 μM) for 3 days. Protein synthesis and RNA replication were analyzed by western blotting and qRT-PCR, respectively. Western blotting was performed using anti-DENV NS2B antibody, anti-HO-1, and anti-GAPDH antibody. GAPDH protein levels showed equal loading of cell lysates. Total cellular RNA was extracted and analyzed by qRT-PCR. The DENV RNA level was normalized by cellular *gapdh* mRNA. “0” indicates treatment with 0.1% DMSO. DENV RNA levels were presented as percentage changes compared to those in parental Huh-7 cells, in which the level was presented as 1. Results are expressed as mean ± SD (error bar) of three independent experiments. *P versus non-SnPP treated or non-specific shRNA transfected control group; #P versus lucidone alone treated control group.

Current studies have revealed that the HO-1 metabolite biliverdin can suppress DENV replication by inhibiting viral protease activity [5]. Lucidone exhibits HO-1 inductive activity against HCV replication [10]. To identify whether induction of HO-1 contributes to the anti-DENV activity of lucidone, we first examined the inductive effect of lucidone on HO-1 expression in the presence of DENV infection using a transient HO-1 promoter activity assay with the HO-1 promoter-driven firefly luciferase expression vector pHO-1-Luc. The results revealed that lucidone concentration-dependently induced DENV-reduced HO-1 promoter activity (Fig. 3A). Furthermore, we analyzed the inductive effect of lucidone on HO-1 expression by qRT-PCR and western blotting. As expected, a concentration-dependent induction of HO-1 RNA (Fig. 3B) and protein (Fig. 3C) levels was observed in lucidone-treated cells. The induced HO-1 protein level was almost comparable to the basal level of HO-1 in parental Huh-7 cells treated with 40 μM lucidone. We then performed a complementary experiment based on suppression of HO-1 expression to examine the inhibitory effect of lucidone-mediated HO-1 induction on DENV replication. Huh-7 cells were transiently transfected with HO-1-specific shRNA at a concentration range of 0.25–2 μg, and then infected with DENV. Subsequently, these DENV-infected cells were treated with 40 μM lucidone for 3 days, and DENV replication efficiency was analyzed by western blotting and qRT-PCR. As shown in Fig. 4A, the increased level of DENV NS2B (upper panel) was comparable to the reduced expression of HO-1 (middle panel), revealing that the reducing HO-1 reversed the inhibitory effect of lucidone on viral protein expression in a concentration-dependent manner (lanes 3–6), compared to that in HO-1 shRNA-untransfected cells in the presence of lucidone (lane 2). Notably, recovery of the NS2B protein level at the high concentration of HO-1 shRNA was comparable to the protein levels in untreated DENV-infected cells (lanes 1 and 6). Consistent with the protein expression results, the recovery percentage of DENV RNA levels correlated to the increased amount of HO-1 shRNA expression compared to that in lucidone-treated DENV-infected cells without HO-1 silencing (Fig. 4B). In addition to the genetic approach, we used SnPP, a specific HO-1 inhibitor, to confirm the role of HO-1 in the anti-DENV action of lucidone. DENV-infected Huh-7 cells were treated with lucidone alone or co-treated with lucidone and SnPP for 3 days. The results demonstrated that DENV protein and RNA levels in the lucidone-treated cells were recovered gradually in response to SnPP (Fig. 4C and 4D). Taken together, these results support our conclusion that upregulation of HO-1 expression contributes to the antiviral activity of lucidone.

### Lucidone significantly reduces DENV NS2B/NS3 protease activity

**Figure 5.**
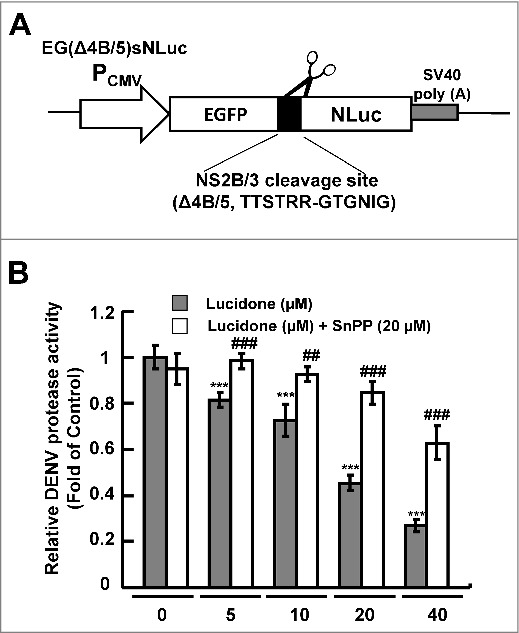
Reduction of DENV protease activity by lucidone. (A) Schematic diagram of the protease reporter vector with DENV NS2B/3 cleavage siteΔ4B/5 (TTSTRR-GTGNIG) between enhanced green fluorescent protein (EGFP) and secretory nano luciferase (pEG(Δ4B/5)sNLuc). (B) Lucidone suppressed DENV protease activity but attenuated with SnPP treatment. Huh-7 cells were co-transfected with pEG(Δ4B/5)sNLuc and NS2B/3 expression vector pNS2B(G_4_SG_4_)NS3. Subsequently, the transfected cells were treated with the indicated concentrations of lucidone in the presence or absence of 20 μM SnPP. After 3 days, the medium and total cell lysates were analyzed for luciferase activity. “0” indicates treatment with 0.1% DMSO.  In each transfection experiment, transfection of firefly luciferase expression vector pCMVLuc served as the transfection control to normalize the nano luciferase activity. The relative DENV protease activity was presented as fold changes relative to that in lucidone-untreated cells, which is defined as 1. Results are expressed as mean ± SD (error bar) of three independent experiments. *P versus non-lucidone treated control group; #P versus equal concentration lucidone without SnPP treated group; ^a^P versus SnPP alone treated group.

Our previous studies showed that the HO-1 metabolite biliverdin inhibits DENV protease activity, leading to an increase in the antiviral IFN responses against DENV infection *in vitro* and *in vivo* [5]. To identify the effect of lucidone-mediated HO-1 induction on DENV protease activity, Huh-7 cells were cotransfected with the NS2B/NS3 protease expression vector pNS2B(G_4_SG_4_)NS3 and its substrate reporter vector pEG(Δ4B/5)sNLuc (Fig. 5A), followed by incubation in various concentrations of lucidone with or without the HO-1 inhibitor SnPP for 3 days, in which luciferase activity reflected DENV protease activity. As shown in Fig. 5B, a concentration-dependent reduction in protease activity was observed in lucidone-treated cells (black columns) compared to its activity in untreated cells. In contrast, treatment with SnPP gradually attenuated the inhibitory effect of lucidone on NS2B/NS3 protease activity (white columns).

### Lucidone significantly increases the IFN-mediated antiviral response *in vitro* and *in vivo*

**Figure 6.**
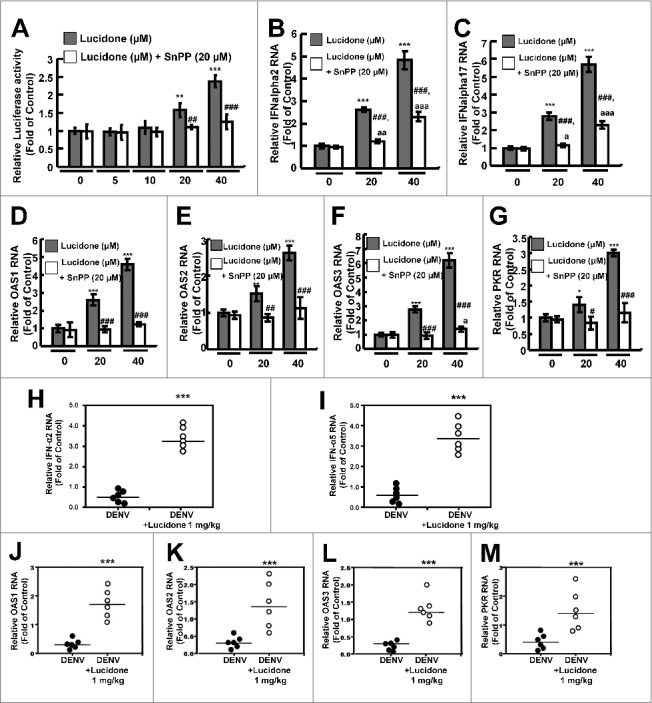
Effect of induction of IFN response by lucidone on the inhibitory effect of DENV replication. Lucidone induced antiviral IFN responses but attenuated with SnPP treatment. (A) DENV-infected Huh-7 cells were transiently transfected with the IFN response reporter vector, pISRE-Luc. Subsequently, the transfected cells were treated with the indicated concentrations of lucidone in the presence or absence of 20 μM SnPP for 3 days, and total cell lysates were analyzed for luciferase activity. (B, C) Total RNA of lucidone-treated cells in the presence or absence of 20μM SnPP was extracted to quantify IFN-2 and IFN-17 levels by qRT-PCR. (D–G) Total RNA of lucidone-treated cells in the presence or absence of 20μM SnPP was extracted to quantify OAS1, OAS2, OAS3, and PKR RNA levels by qRT-PCR. (H-M) 0.1g of brain tissue of DENV-infected ICR suckling mice was collected and the total RNA was extracted to quantify IFN-2, IFN-5, OAS1, OAS2, OAS3 and PKR levels by qRT-PCR. Relative RNA levels were normalized by cellular *gapdh* mRNA levels. “0” indicates treatment with 0.1% DMSO. The RNA levels were presented as fold changes compared to those in lucidone-untreated cells, in which the level was presented as 1. Results are expressed as mean ± SD (error bar) of three independent experiments. *P versus DENV-infected control group; #P versus equal concentration lucidone without SnPP treated group; ^a^P versus SnPP alone treated group.

A previous report demonstrated that DENV NS2B/NS3 protease disrupts the human mediator of IRF3 activation (MITA)-mediated antiviral interferon response to facilitate viral propagation [13]. Because DENV NS2B/NS3 protease activity was significantly suppressed by lucidone, we further verified whether lucidone activated the DENV-suppressed antiviral IFN response to inhibit DENV replication. We first performed a transient ISRE activity assay using the ISRE-mediated firefly luciferase expression vector. DENV-infected Huh-7 cells were transfected with pISRE-Luc reporter plasmids and then incubated with different concentrations of lucidone with or without SnPP for 3 days. As shown in Fig. 6A, lucidone treatment significantly increased the ISRE-mediated luciferase activity at the effective antiviral concentrations (black columns) but the elevated activities were abrogated by the SnPP treatment (white columns). In addition, we further measured the antiviral gene expression, including IFN-α2 and -α17 in lucidone-treated DENV-infected cells (20 and 40 μM) by qRT-PCR analysis. As shown in Fig. 6B and 6C, lucidone dramatically increased the mRNA levels of both IFN genes (black columns). In contrast, SnPP treatment attenuated lucidone-activated IFN gene expression (white columns). Under the same experimental conditions, the mRNA levels of IFN-mediated antiviral genes, including protein kinase R (PKR) and 2′-5′-oligoadenylate synthetase 1 (OAS1), OAS2, and OAS3 were analyzed by qRT-PCR. As expected, lucidone significantly induced antiviral gene expression (Fig. 6D–F, black columns) compared to the expression in untreated cells. In contrast, treatment with SnPP attenuated the induction response of lucidone on antiviral IFN gene expression (Fig. 6D–F, white columns). IFN gene expression and IFN-mediated antiviral gene expression were further examined *in vivo* using the DENV-infected ICR mice model. The brain tissues of DENV-infected ICR mice were harvested for qRT-PCR analysis. As shown in Fig. 6H and 6I, the expression of IFN-α2 and -α5 in the lucidone-treated DENV-infected group were induced compared to those in the untreated group. Furthermore, lucidone significantly induced the IFN-mediated antiviral gene expression, including PKR, OAS1, OAS2, and OAS3, in the lucidone-treated DENV-infected group compared to that in the untreated group (Fig. 6J–6M). Based on these *in vitro* and *in vivo* results, we concluded that lucidone treatment suppressed DENV NS2B/NS3 protease by inducing HO-1, and then the DENV-suppressed antiviral IFN response was rescued to inhibit DENV replication.

### Nrf2-mediated HO-1 induction is involved in anti-DENV activity of lucidone.

**Figure 7.**
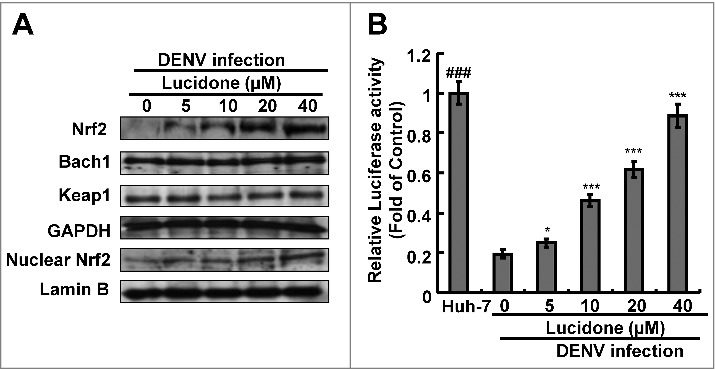
Effect of lucidone on the expression of Nrf2 in DENV-infected cells. Lucidone activated Nrf2 expression and the efficacy of nucleus translocation. (A) DENV-infected Huh-7 cells were treated with the indicated concentrations of lucidone. The cell lysate was separately extracted from the cytoplasm and nuclear extracts and analyzed by western blotting using anti-Bach1, anti-keap1, anti-Nrf2, anti-GAPDH, and anti-LaminB1 antibodies. GAPDH and Lamin B protein levels showed equal loading of cell lysates. (B) DENV-infected cells were transfected with reporter plasmid p3xARE-Luc and treated with the indicated concentrations of lucidone for 3 days, and total cell lysates were analyzed for luciferase activity. “0” indicates treatment with 0.1% DMSO. The luciferase activity was presented as fold changes compared to that in lucidone-untreated cells, in which the level was presented as 1. Results are expressed as mean ± SD (error bar) of three independent experiments. *P versus lucidone non-treated control group; #P versus DENV-infected control group.

**Figure 8.**
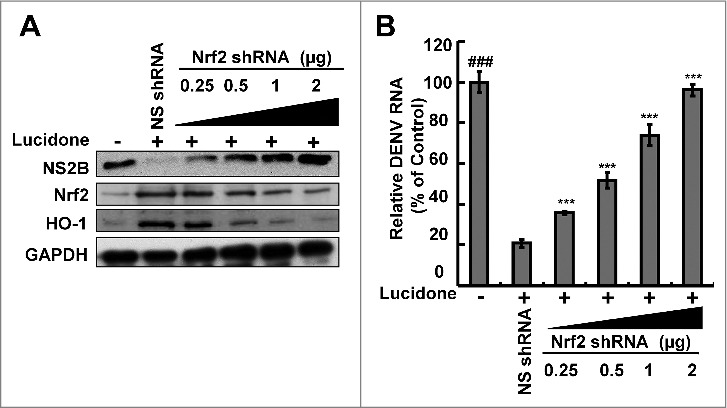
Restoration of DENV protein synthesis and RNA replication by inhibition of Nrf2 expression in lucidone-treated cells. Silencing Nrf2 expression attenuated the induction of HO-1 and the antiviral activity of lucidone. DENV-infected cells were transfected with different amounts (0–2 µg) of Nrf2-specific shRNA and treated with or without 40 µM lucidone for the detection of (A) DENV protein synthesis and (B) DENV RNA replication after incubation for 3 days. Western blotting was performed using anti-DENV NS2B, anti-Nrf2, anti-HO-1, and anti-GAPDH antibodies. GAPDH protein levels showed equal loading of cell lysates. Total cellular RNA was extracted and analyzed by qRT-PCR. The ratio of DENV RNA level was normalized by cellular *gapdh* mRNA level. “0” indicates treatment with 0.1% DMSO. The RNA levels were presented as percentage changes compared to those in lucidone-untreated cells, in which the level was presented as 100%. Results are expressed as mean ± SD (error bar) of three independent experiments. *P versus non-specific shRNA transfected control group; #P versus DENV-infected control group.

Three important nuclear factors, including Nrf2, keap1, and Bach1, transcriptionally mediate HO-1 expression [14,15]. To examine which nuclear factor was responsible for HO-1 induction by lucidone, we first analyzed the effect of lucidone on the protein expression of the nuclear factors in DENV-infected cells. As shown in Fig. 7A, lucidone increased total Nrf2 protein levels and nuclear Nrf2 accumulation in a concentration-dependent manner. We further used a p3xARE-Luc luciferase reporter construct containing three copies of Nrf2-dependent ARE to confirm the specific transactivation of Nrf2-mediated HO-1 expression. The p3X-ARE-Luc-transfected Huh-7 cells were infected with DENV, followed by incubation with increasing concentrations of lucidone for 3 days. As shown in Fig. 7B, lucidone enhanced the luciferase activity in a concentration-dependent manner, revealing that lucidone-upregulated Nrf2 contributes to HO-1 induction. To further investigate whether the inhibitory effect of lucidone on DENV replication depended on Nrf2 gene expression, Huh-7 cells were transiently transfected with Nrf2 specific shRNA at different concentrations (0.25–2 μg) and were then infected with DENV. Subsequently, these cells were treated with 40 μM lucidone for 3 days, and the protein synthesis activities of DENV, Nrf2, and HO-1 were analyzed by western blotting. As shown in Fig. 8A, the increased protein level of DENV NS2B (upper panel) was comparable to the reduced expression of Nrf2 and HO-1 (middle panel). Similar results were observed in the qRT-PCR analysis (Fig. 8B). Taken together, these results reveal that the antiviral action of lucidone was dependent on Nrf2-mediated HO-1 induction.

## Discussion

Several studies have elucidated various aspects of DENV-induced disease, including reactive oxygen species (ROS) and inflammation [4,16]. However, the pathogenesis of the disease is complex and remains unclear. ROS-related innate immunity and inflammatory pathways are activated at the early stages of DENV infection, leading to an imbalance in redox homeostasis, which suggests a link with DHF/DSS progression in DENV-infected patients [17,18]. DENV-induced ROS levels or ROS-associated molecules are considered as useful biomarkers to predict disease severity [19]. In a previous study, glutathione (GSH), a major water-soluble antioxidant, decreased in the serum of DENV-infected patients. In contrast, supplemental GSH markedly reduced DENV production and viral-induced liver and brain injury [20]. In our study, the antioxidant lucidone also served as a protective agent in a DENV-induced life-threatening animal model and reduced viral replication (Figs. 1 and 2). Therefore, antioxidant treatment is considered as a potential strategy against either viral infection or infection-associated symptoms.

Activation of inflammatory signaling pathways and antiviral responses are correlated with the production of ROS [21–24]. The antioxidant response is one of the antimicrobial responses of cells against a pathogenic infection [25]. HO-1 is an important antioxidant that responds to virus-induced oxidative stress to maintain tissue homeostasis [26–28]. Previous studies have reported that activation of HO-1 by the antioxidant molecule andrographolide efficiently inhibit DENV replication and protect mice from life-threatening DENV infection *in vitro* and *in vivo* [5]. In addition, we also found that lucidone significantly induced HO-1 expression in Huh-7 parental cells (Supplementary Fig. S3). Lucidone upregulated DENV-reduced HO-1 expression and exhibited anti-DENV activity through an HO-1-dependent pathway (Figs. 3 and 4). We previously verified that the HO-1 metabolite biliverdin inhibits DENV NS2B/NS3 protease activity and suggested that preventing cleavage of the IFN adaptor MITA/STING by NS2B/NS3 protease could activate the antiviral IFN signaling pathway against DENV replication. We observed that lucidone consistently suppressed DENV NS2B/NS3 protease activity and stimulated the antiviral IFN responses *in vitro* and *in vivo*, which were dependent on HO-1 activity (Figs. 5B and 6). NS3 catalytic activity is inhibited by lucidone, whereas the antiviral effects of lucidone may not only come from inducing the antiviral IFN response but also by suppressing viral polyprotein processing through inhibition of NS2B/3 function. We propose that there is a cumulative or synergistic antiviral effect of lucidone due to inhibition of bifunctional NS2B/3 protease. Therefore, lucidone could be a potential agent against pathogenic infection through activation of antioxidant responses.

Innate antiviral immune responses and cellular apoptosis are regulated by intracellular ROS, which stimulate a variety of endogenous antioxidant enzymes [19,29,30]. Cells induce a variety of cytoprotective enzymes during ROS detoxification, including HO-1 [31,32]. These genes encode a common prompter element ARE that is transactivated by Nrf2 [33]. Previous studies have shown that silencing Nrf2 reduces the antiviral response and suppresses replication of respiratory syncytial virus and influenza virus [34,35]. Influenza virus infection also induces the secretion of inflammatory cytokines and exacerbates lung injury in Nrf2-deficient mice [36]. Silencing Nrf2 consistently improves viral replication and ROS levels after DENV challenge [19]. Therefore, Nrf2 is considered as a regulator of limiting tissue damage and pathogenic infection [37]. Herein, we found that lucidone induced HO-1 promoter activity and mRNA and protein levels were associated with Nrf2 activation upon DENV infection (Fig. 7), which is consistent with previous studies showing that lucidone activated Nrf2 in UVA-irradiated human skin keratinocytes and HCV replicon cells [10,38]. In addition, we found that the Nrf2 and HO-1 knockdown had no significant effects on DENV infectivity and that the antiviral activity of lucidone was dependent on Nrf2-mediated HO-1 induction (Supplementary Fig. S4 and Fig. 8). Therefore, we propose that lucidone possesses not only anti-DENV activity but also plays a protective role against DENV-induced ROS production. However, the Nrf2/ARE-mediated HO-1 expression can be regulated by multiple upstream kinases, such as mitogen activated protein kinase (MAPK), phosphoinositide 3-kinase (PI3K), Akt, protein kinase C (PKC), and glycogen synthase kinase 3 beta, to maintain cellular redox homeostasis [39]. Because the relationship between Nrf2 expression and DENV replication remains unclear, it is necessary to elucidate the specific signaling pathways involved in the anti-DENV activity of lucidone.

Natural products are considered a source for discovering and developing new pharmaceuticals through synthetic modification. A number of plants exhibit anti-DENV activity [40]. However, few investigations have reported the targets or detailed mechanisms of these antivirals against viruses. In addition to viral proteins, some host factors or signaling pathways required for viral replication were considered a potential antiviral mechanism. Some studies have shown that cavinafungin, targeting a host signal peptidase, and NITD-982, containing an isoxazole-pyrazole core structure targeting pyrimidine biosynthesis, exhibit anti-DENV activity with EC_50_ value of 5 nM and 2.4 nM, respectively [41,42]. Although those inhibitors were more potent than lucidone against DENV *in vitro*, they lack confirmation of *in vivo* effectiveness. Here, we confirmed the anti-DENV activity of lucidone *in vivo* (Fig. 1) and further demonstrated the molecular mechanism of lucidone against DENV by targeting cellular HO-1 induction (Fig. 4). Previous studies also demonstrated that inhibitors targeting COX-2 and tyrosinase exhibit significant anti-DENV replication [43,44]. Additionally, lucidone exhibits anti-COX-2 and anti-tyrosinase activities in macrophages and melanoma cells, respectively [45,46]. It will be worth investigating whether inhibiting COX-2 or tyrosinase contributes to anti-DENV activity of lucidone *in vitro* and *in vivo.*

In past decades, some reports have isolated virus particles from brain tissue or cerebrospinal fluid of patients with DENV [47–49]. Many syndromes are involved in dengue encephalopathy, such as prolonged shock, hyponatremia, hepatic failure, and intracranial bleeding [50, 51]. According to the dengue guidelines endorsed by the World Health Organization (WHO) in 2009, central nervous system involvement is one of the criteria for classifying severe dengue [52]. The ICR suckling mice model has been reported to assess central nervous system symptoms after intracranial inoculation of DENV because severe neurological symptoms and death are observed in this disease [53]. A substitutive model has also been used for studies of virus-host interactions and antiviral drug validation [5,54–56]. However, the inoculation route does not accurately mimic natural infection. Numerous animal models have been established for different purposes, such as vaccine development, pre-clinical antiviral drug test, or dengue pathogenesis [57]. For example, 129/Sv genetic background (AG129) mice, which are deficient in IFN-α/β and -γ receptors, support robust levels of DENV replication after subcutaneous inoculation of DENV [58]. AG129 mice have been widely used to study DENV-related neurodisease, the antibody response, systemic infection, lymphopenia, vascular leakage, liver and intestinal damage, the immune response, and even vaccine development [59]. The AG129 model has also been used in antibody-dependent enhancement studies, which contributed pre-existing, subneutralizing antibodies to increase DENV infection [60]. To further investigate the activity of lucidone against DENV-induced pathogenesis, we plan to identify the protective efficacy of lucidone in DENV-infected AG129 mice by administering oral or intraperitoneal injections to validate the inhibitory effect on viremia or plasma leakage in the future. Based on our studies, we propose that lucidone may not only significantly reduce the DENV viral titer but also protect mice from DENV-induced syndrome, which will further reveal the safety and practicality of lucidone in anti-DENV therapeutic development.

**Figure 9.**
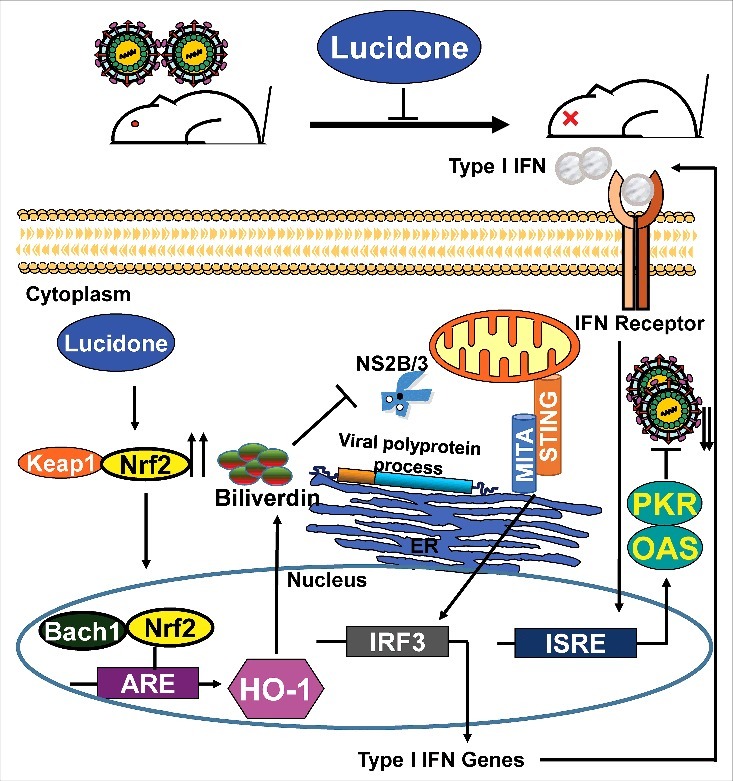
Model of action of lucidone against DENV replication Lucidone induces Nrf2-mediated HO-1 signaling pathway against DENV replication through the inhibition of NS2B/3 protease activity and the induction of antiviral IFN response, ultimately extending the life-span of DENV-infected mice.

To summarize, we demonstrated that lucidone not only possesses a protective effect against DENV-induced lethality and illness but also exerts a significant anti-DENV activity by inhibiting NS3 protease activity for the induction of antiviral IFN responses through the Nrf2/HO-1 signaling pathway (Fig. 9). Furthermore, our data support that strategies aimed at activating HO-1 may be a valid therapeutic avenue due to its ability to inhibit dengue replication while concomitantly reducing the symptoms associated with DENV-induced disease.

## Supplementary Material

KVIR_A_1421893_supp.zip
