# Supplementary material for: Lucidone suppresses dengue viral replication through the induction of heme oxygenase-1
Source: Virulence. 2018 Feb 27;9(1):588–603. doi: 10.1080/21505594.2017.1421893 (PMC5955471; doi:10.1080/21505594.2017.1421893)
Supplement: KVIR_A_1421893_supp.zip [file kvir-09-01-1421893-s001.zip › KVIR_A_1421893 supp/2017VIRULENCE0122R2-file002.doc]

**Table 1.** Oligonucleotide sequences for real-time RT-PCR

| **Oligonucleotide** **Name** | **Sequence 5**'**-3**' |
| --- | --- |
| DENV serotypes |  |
| 5' NS5 (Type1) | 5'-CAGGTCAAACGCAGCTATTG |
| 3' NS5 (Type1) | 5'-CCACTCCACTGAGTGAATTC |
| 5' NS5 (Type2) | 5'-TGTATGCCGATGACACCGCA |
| 3' NS5 (Type2) | 5'-TCTTTGCACACGGACCACCT |
| 5' NS5 (Type3) | 5'-TCAGAACTAACGCAGCCATG |
| 3' NS5 (Type3) | 5'-AGAGTTTTCACGCGAGAACC |
| 5' NS5 (Type4) | 5'-AGATCAAACGCAGCCATAGG |
| 3' NS5 (Type4) | 5'-CTTCCACTCCACTCCATGAA |
| Human factors |  |
| 5' GAPDH | 5'-GTCTTCACCACCATGGAGAA |
| 3' GAPDH | 5'-ATGGCATGGACTGTGGTCAT |
| 5'OAS1 | 5’- CAAGCTTAAGAGCCTCATCC |
| 3'OAS1 | 5’- TGGGCTGTGTTGAAATGTGT |
| 5'OAS2 | 5’- ACAGCTGAAAGCCTTTTGGA |
| 3'OAS2 | 5’- GCATTAAAGGCAGGA AGCAC |
| 5'OAS3 | 5’- CACTGACATCCCAGACGATG |
| 3'OAS3 | 5’- GATCAGGCTCTTCAGCTTGG |
| 5'PKR | 5’- ATGATGGAAAGCGAACAAGG |
| 3'PKR | 5’- GAGATGATGCCATCCCGTAG |
| 5'IFN-α2 | 5’- GCAAGTCAAGCTGCTCTGTG |
| 3'IFN-α2 | 5’- GATGGTTTCAGCCTTTTGGA |
| 5'IFN-α17 | 5'-AGGAGTTTGATGGCAACCAG |
| 3'IFN-α17 | 5'-CATCAGGGGAGTCTCTTCCA |
| Mouse factors |  |
| 5' GAPDH | 5'-CCATGCCATCACTGCCACCC |
| 3' GAPDH | 5'-GCCATGCCAGTGAGCTTCCC |
| 5'OAS1 | 5'-GGACCCCGCTGACCCAACAA |
| 3'OAS1 | 5'-CAACCTCCGTCGGCACCTCC |
| 5'OAS2 | 5'-GGTGGGTCTTCAGGGGTGCC |
| 3'OAS2 | 5'-GCAGCAGGTCCCAGATGGCA |
| 5'OAS3 | 5'-CCGTGCCTGGACTGAGCCTC |
| 3'OAS3 | 5'-GGCTGAGGTTTGGTGCCGGA |
| 5'PKR | 5'-AGAGCCCGCCGAAAACTGCC |
| 3'PKR | 5'-CGCTGTTAAACCTGGCGTCCA |
| 5'IFN-α2 | 5'-TGACCTCCACCAGCAGCTCA |
| 3'IFN-α2 | 5'-TCTGCTCTGACCACCTCCCA |
| 5'IFN-α5 | 5'-CCATCCCTGTCCTGAGTGAGCT |
| 3'IFN-α5 | 5'-AGATTCCTGCACCCCGACCT |
